# Deep learning approach for automatic landmark detection and alignment analysis in whole-spine lateral radiographs

**DOI:** 10.1038/s41598-021-87141-x

**Published:** 2021-04-07

**Authors:** Yu-Cheng Yeh, Chi-Hung Weng, Yu-Jui Huang, Chen-Ju Fu, Tsung-Ting Tsai, Chao-Yuan Yeh

**Affiliations:** 1 Department of Orthopaedic Surgery, Spine Division, Bone and Joint Research Center, Chang Gung Memorial Hospital and Chang Gung University College of Medicine, Taoyuan, Taiwan, ROC; 2 aetherAI Co., Ltd., 9F., No.3-2, Yuanqu St., Nangang Dist., Taipei City, 115 Taiwan, ROC; 3 Department of Medical Imaging and Intervention, Chang Gung Memorial Hospital and Chang Gung University College of Medicine, Taoyuan, Taiwan, ROC

**Keywords:** Skeleton, Machine learning, Radiography

## Abstract

Human spinal balance assessment relies considerably on sagittal radiographic parameter measurement. Deep learning could be applied for automatic landmark detection and alignment analysis, with mild to moderate standard errors and favourable correlations with manual measurement. In this study, based on 2210 annotated images of various spinal disease aetiologies, we developed deep learning models capable of automatically locating 45 anatomic landmarks and subsequently generating 18 radiographic parameters on a whole-spine lateral radiograph. In the assessment of model performance, the localisation accuracy and learning speed were the highest for landmarks in the cervical area, followed by those in the lumbosacral, thoracic, and femoral areas. All the predicted radiographic parameters were significantly correlated with ground truth values (all *p* < 0.001). The human and artificial intelligence comparison revealed that the deep learning model was capable of matching the reliability of doctors for 15/18 of the parameters. The proposed automatic alignment analysis system was able to localise spinal anatomic landmarks with high accuracy and to generate various radiographic parameters with favourable correlations with manual measurements.

## Introduction

Spinal curvature was modified in the human species, as one of the few bipedal animals, to enable horizontal gaze while both hands are free for performing complex tasks. Lordotic curvature was thus developed in the cervical and lumbar vertebrae to maintain the centre of mass within the area of both feet (stance width). This concept was further elaborated by ‘conus of economy’ theory proposed by Dubousset^1^: shifting the centre of mass away from the standing area would result in additional energy expenditure. Various radiographic parameters have been developed and validated on standard uniplanar two-dimensional (2D) whole-spine radiographs to evaluate spinopelvic harmony and the sagittal balance of the spine^2^.

In the clinical setting, manual measurement and calculation of numerous spinopelvic parameters on whole-spine radiographs require substantial time and effort. Thus, semi-automated or automated spine radiographic anatomic landmark localisation and vertebral segmentation on plain radiographs have been studied for over a decade^3,4^. Recently, deep learning methods have been applied to automatic sagittal radiographic parameter measurement, with mild to moderate standard errors and favourable correlations with manual measurement^5–9^. It is worth mentioning that some approaches can better characterise spinal alignment, as they can estimate several spinopelvic parameters at once. For example, Galbusera et al*.*^8^ trained 78 distinct deep learning models to derive 78 landmark coordinates and six spinopelvic parameters. Korez et al.^5^ were able to estimate five spinopelvic parameters following a detection-based approach, which detects four anatomic structures first and then regresses five anatomic landmarks within the detected structures later. However, these approaches still have room for improvement:

1. The detection-based model may fail to distinguish between similar adjacent anatomic structures (e.g. the third and fourth thoracic vertebra).
2. The predicted radiographic parameters are not comprehensive enough to cover the whole spinal column and pelvic structures.
3. The coordinate regressors in some studies^5,10^ may lose the ability to utilise all relevant anatomic structures of the entire image, as their approach involves the split of images into small patches.
4. The test datasets are often insufficient in quantity and diversity for spinal pathologies, and they might not represent real clinical situations.

Our present study addresses these problems by using an ensemble of two end-to-end trainable models to localise 45 anatomic landmarks on whole-spine lateral radiographs. We created a dataset consisting of 2210 radiographs, the largest, annotated dataset with various spinal pathologies to date. Using our dataset, we trained deep learning models which can predict landmark coordinates using anatomic structures of the entire radiographs. Our models were able to find 45 anatomic coordinates of the whole spinal column and pelvic structures with low median error and generate various radiographic parameters with favourable correlations with manual measurements.

## Results

### Study design

**Figure 1:**
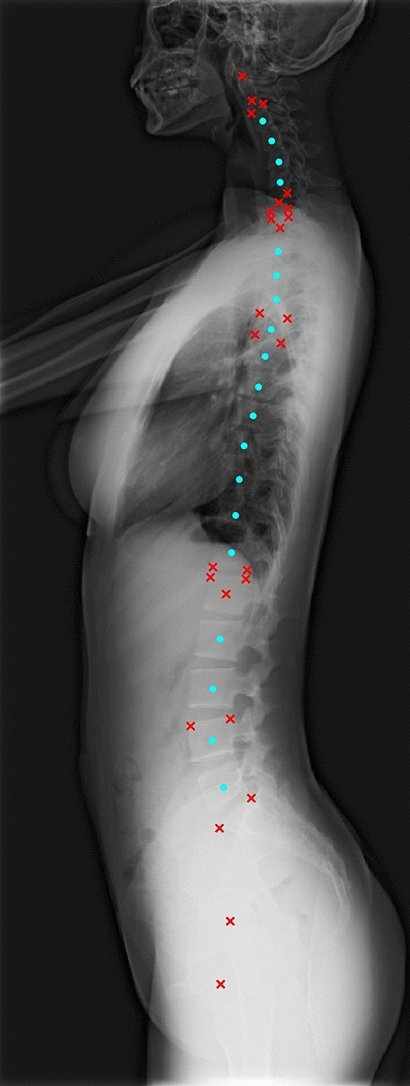
Annotated anatomic landmarks. 45 anatomic landmarks were annotated on a whole-spine lateral radiograph. 26 landmarks related to spinopelvic parameters are highlighted by crosses (in red); the other landmarks, which are at vertebral centres and can be used to determine the spinal curve, are highlighted by solid circles (in cyan). Detailed descriptions for each landmark are presented in the Methods section.

Whole-spine plain radiographs are widely used as the first-line examination for standing patients with scoliosis, kyphosis, spinal imbalance, or patients who have received long spinal instrumentation. This study aims to automatically annotate 45 landmark coordinates (Fig. 1) on whole-spine lateral images. With the derived landmark coordinates, 18 spinopelvic parameters (Supplementary Fig. S1) can be used to (1) evaluate whole spinopelvic alignment and balance in standing positions; (2) perform postoperative follow-ups for implants across multi-level and wide regions.

**Figure 2:**
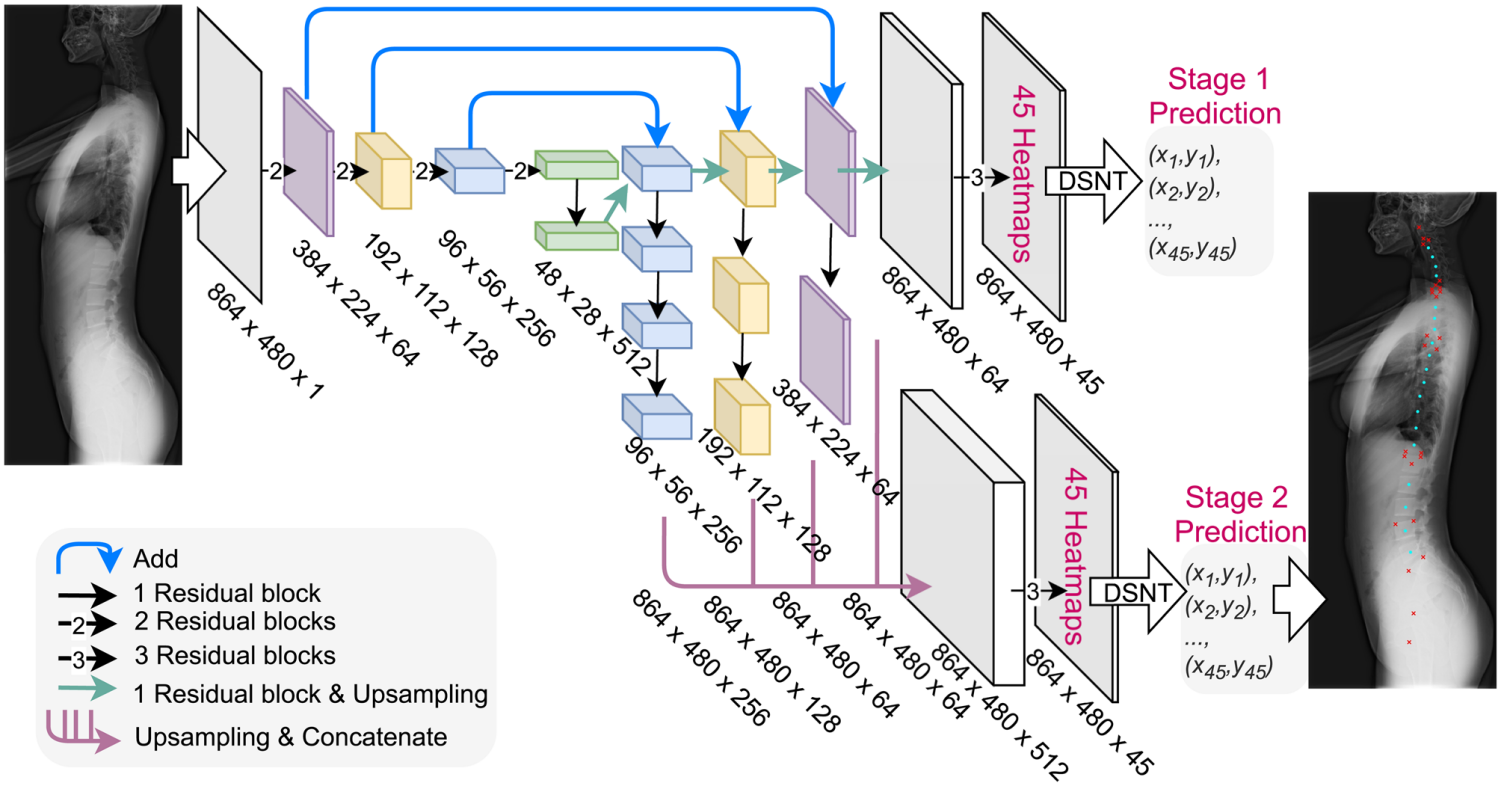
Model architecture. The radiographs were down-sampled to $864\times 480$ px before being fed into the network. The network contained 25 trainable ‘residual blocks’. Each ‘residual block’ comprised two consecutive $3\times 3$ convolutional layers if its input and output had the same shape (if not, an additional $1\times 1$ convolutional layer was added to its skip-connection part). The network also contained three types of nontrainable blocks (‘Add’, ‘Concatenate’, and ‘Upsampling’), which were used for tensor addition, concatenation, and bilinear upsampling. The network had two stages. At the end of each stage, 45 landmark coordinates were predicted by applying ‘DSNT’ (illustrated in the Methods section) to the feature maps (heatmaps) of each landmark. In addition, the stage 1 prediction was an intermediate result used during training and discarded during inference.

**Figure 3:**
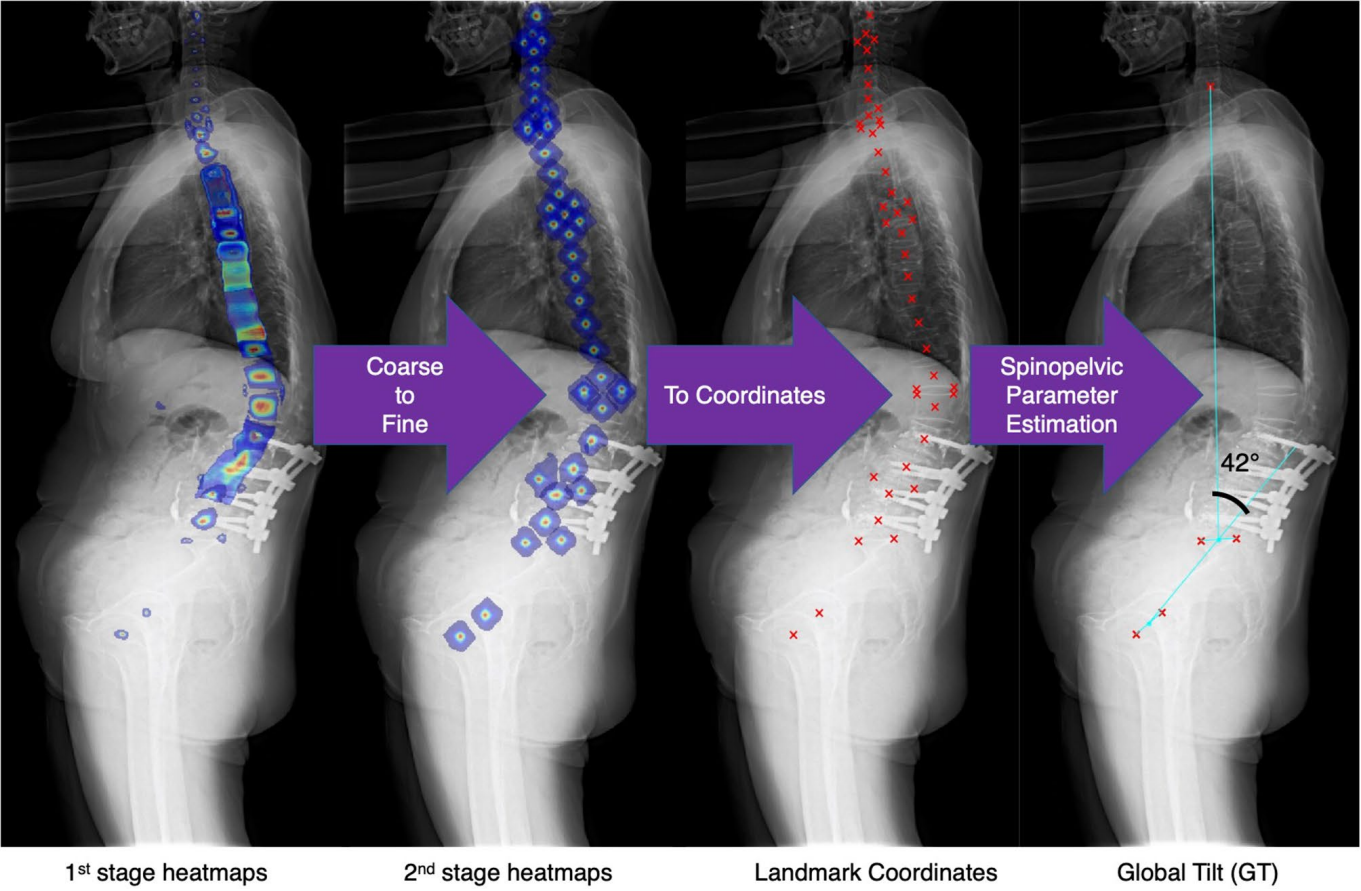
Automatic spine parameter estimation process. From the first to the second stage of the network, heatmaps of 45 landmarks were predicted from coarse to fine. Finally, the predictions of landmark coordinates were extracted from the fine (i.e. second stage) heatmaps. The predicted locations were then postprocessed to obtain 18 spine parameters (here, we plot ‘Global Tilt’ as an example).

**Figure 4:**
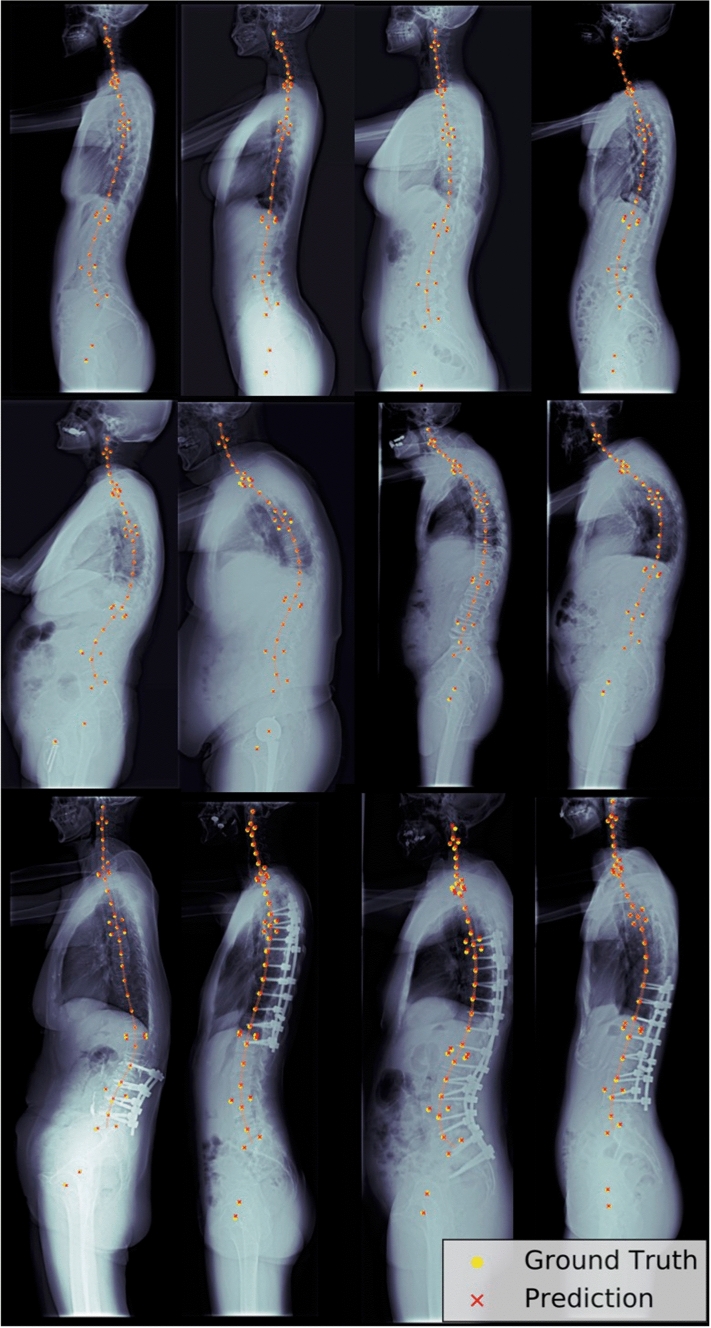
Prediction examples. Examples of model predictions in images with different spinal pathologies, i.e. scoliosis, kyphosis, or implant. For each radiograph, the predicted locations (red crosses) and human-annotated locations (yellow circles) were plotted. We also approximated the spinal curves (orange line) by interpolating the predicted locations of vertebral centres.

To estimate 45 landmark coordinates, we developed our deep learning model (Fig. 2) based on Cascaded pyramid Network (CPN)^11^. Our model differs from the original CPN since (1) we added Differentiable Spatial to Numerical Transform (DSNT)^12^ layers so that the landmark coordinates can be regressed directly; (2) similar to the original CPN, we used 2D heatmaps (probability density maps) to indicate the probable locations of landmarks. However, we added an additional regularisation loss on heatmaps (as illustrated in the Methods section) so that our model can predict heatmaps of landmarks with arbitrary shapes and sizes at the first stage and with small splotches of constraint shapes (narrow Gaussian or narrow exponential^6^) at the second stage. Our deep learning model can thus localise the anatomic landmarks in a two-stage, coarse-to-fine manner. The predicted 45 landmark coordinates (Fig. 1) can then be used to estimate spinopelvic parameters using our computer program developed in Python. A schematic diagram of the pipeline for parameter estimation is illustrated in Fig. 3. Some examples of landmark predictions are shown in Fig. 4.

Our method can estimate 18 spinopelvic parameters automatically, including:

- 5 fundamental spinopelvic parameters^13–16^: pelvic incidence (PI), sacral slope (SS), pelvic tilt (PT), lumbar lordosis (LL), and sagittal vertical axis (SVA).
- 8 regional spinal parameters^17–22^: cervical lordosis (CL), T1 slope (T1S), cervical SVA (cSVA), global thoracic kyphosis (GTK), T1–T5 proximal thoracic kyphosis (PTK), T5–T12 main thoracic kyphosis (MTK), L4-S1 lordosis (L4SL), and lumbar pelvic angle (LPA).
- 5 global spinopelvic parameters^23–27^: spino-sacral angle (SSA), global tilt (GT), T1-pelvic angle (TPA), C7 plumb line/sacrofemoral distance ratio (C7/SFD ratio or Barrey index), and odontoid hip axis (OD-HA).

### Dataset demographics

From January 2018 to April 2020, a total of 2900 consecutive whole-spine lateral plain radiographs were reviewed and annotated under the approval of the institutional review board of our hospital (IRB No. 202000623B0). After excluding (1) 174 images with inadequate length, meaning they did not include either C2 dens or both femoral heads, (2) 294 images with anatomic variance, in which the vertebral column numbered more or fewer than 25 vertebrae, and (3) 222 images with poor contrast preventing identification of pelvic anatomic structures, a total of 2210 images were included in our study. The mean age was 36.3 ± 25.2 (range: 2–96) years at the time radiographs were taken. The dataset was divided into three categories, namely scoliosis (1041 images), kyphosis (466 images), and implant (703 images), according to the disease aetiologies or the presence of one or more implants. For the images with spinal implants, the mean fixation length was 8.1 ± 3.8 (range: 2–18) levels, with the upper instrumented vertebrae ranging from C4 to L5 and the lower instrumented vertebrae ranging from C7 to the ilium.

### Learning speed for landmarks in different spinal areas

**Figure 5:**
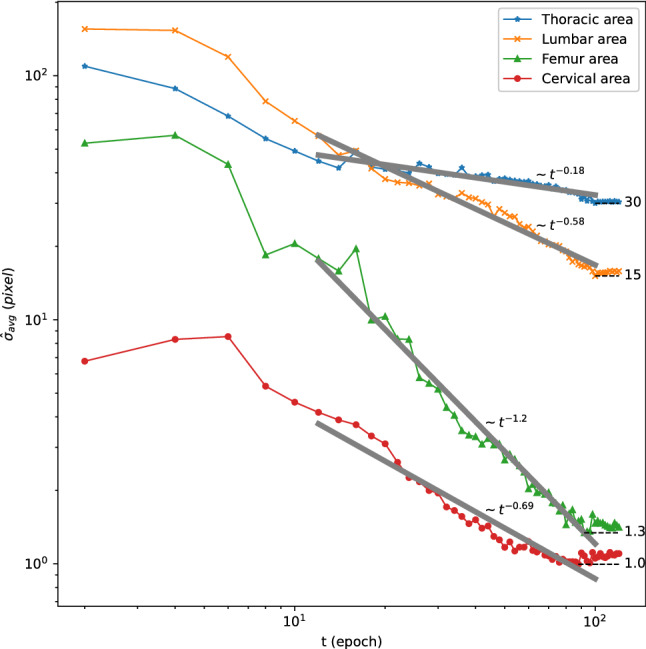
Learning speed of landmarks in different spinal areas. We categorised the landmarks of vertebral centres and femoral heads into four areas (cervical, thoracic, lumbar, and femoral heads) and plotted the per-area averaged standard deviation (SD) of the first-stage heatmaps against the training time (epoch), in log–log scale. From epoch 10 to epoch 100, we observed that the profiles of SDs had a tendency to decay as ${t}^{-\gamma }$. We fitted the decaying profiles in log–log scale (grey solid lines) and estimated $\gamma =0.18$ (thoracic area); $\gamma =0.58$ (lumbar area); $\gamma =0.69$ (femoral heads); and $\gamma =1.2$ (cervical area). For all fitted results, the adjusted ${R}^{2}>0.86$ and $p$ value $< {10}^{-4}$.

We observed that the width of coarse heatmaps produced at the 1^st^ model stage gradually became narrower during training, indicating the neural network became more and more confident about the estimated landmark coordinates. To visualise this narrowing behaviour, we categorised the landmarks of vertebral centres and femoral heads into four areas (cervical, thoracic, lumbar, and femoral heads) and estimated the standard deviations (SDs) of per-landmark first-stage heatmaps. As indicated by Fig. 5, we calculated the per-area averaged SDs and observed that the decay profile of SDs followed the tendency of ${t}^{-\gamma }$, where $t$ is the training time (epoch) and $\gamma$ is the decay rate. The decay rate $\gamma$ can be interpreted as the learning speed for landmarks. We observed that the learning speed for landmarks in the thoracic area was the slowest ($\gamma \approx 0.18$), whereas the learning speed for landmarks in the femoral heads was the fastest ($\gamma \approx 1.2$).

### Performance of the deep learning model for automatic landmark localisation

**Figure 6:**
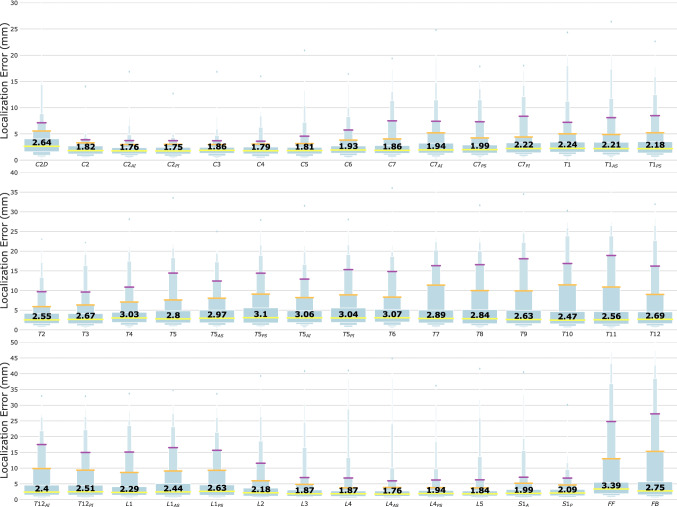
Model performance. Forty-five landmark localisation errors (Euclidean distance between human-annotated and machine-predicted coordinates) were visualised with boxenplots. The horizontal lines in yellow, orange and purple represented 0.5, 0.875, and 0.9375 error quantile, respectively. The value of error median (0.5 quantile) was also displayed using bold-font digits within each boxenplot.

We used localisation error as the metric for performance evaluation on 400 test images. The localisation error was defined as the Euclidean distance between the landmark coordinates of the ground truth and the deep learning model. Due to the non-normal distribution characteristics of the localisation errors, we illustrated the localisation errors of 45 landmarks using boxenplots (also known as letter-value plots^28^), as in Fig. 6. A boxenplot describes error distribution using quantiles. For example, the widest box covers the range of 0.25 to 0.75 quantile (also known as the interquartile range [IQR]); the second-widest box covers the range of 0.125 to 0.875 quantile; and the third-widest box covers the range of 0.0625 to 0.9375 quantile.

The performance of the deep learning model was the highest in the cervical area, where the median localisation errors ranged from 1.75 to 2.64 mm, followed by model performance in the lumbosacral area, with median localisation errors ranging from 1.76 to 2.63 mm. The thoracic area had greater localisation errors than did the cervical and thoracic areas, with median errors ranging from 2.21 to 3.07 mm. Localisation errors were the greatest at the centres of both femoral heads, with median errors of 2.75 mm and 3.39 mm.

We further examined the error distributions of anatomic landmarks using the calculated boxenplots. Error distributions for landmarks in the cervical area were generally narrower (all heights of the third-widest boxes were $<8 \mathrm{mm}$) with shorter tails (most heights of the fourth- to seventh-widest boxes were $<20 \mathrm{mm}$), which indicated accurate predictions of anatomic landmarks. Error distributions for landmarks in the thoracic area were wider (all heights of the third-widest boxes were $<20 \mathrm{mm}$) and longer-tailed (most heights of the fourth- to seventh-widest boxes lay between $10 \mathrm{mm}$ and $30 \mathrm{mm}$), which indicated larger numbers of localisation errors and higher possibility of incorrect level recognition. Model performance was higher in the lumbosacral area; error distributions of landmarks become narrow again below L3 (the heights of the third-widest boxes were $<8 \mathrm{mm}$) but with long tails (heights of the fourth- to seventh-widest boxes ranged from $7 \mathrm{mm}$ to $40 \mathrm{mm}$). For the centres of both femoral heads, the localisation errors were the greatest among all anatomic landmarks; the error distributions of the landmarks were the widest (the heights of the third-widest boxes were $<28 \mathrm{mm}$) and had the longest tails (the heights of the fourth- to seventh-widest boxes were $<50 \mathrm{mm}$).

### Performance of the deep learning model for spinopelvic parameter estimation

Spinal radiographic parameters and prediction errors of the test dataset ($400$ images) are presented in Table 1. Median parameter errors with IQR are presented in addition to mean parameter errors with SD due to the non-normal distribution characteristics of parameter errors. All the predicted radiographic parameters were significantly correlated with ground truth values (all $p < 0.001$).

For fundamental spinopelvic parameters, the mean errors ranged from $1.1^\circ$ (PT) to $5.1^\circ$ (LL), and the median errors ranged from $0.6^\circ$ (PT) to $3.0^\circ$ (LL). No significant differences were observed between the predictions and the ground truth values, with all $p > 0.05$ in Wilcoxon signed-rank tests. The predicted PT and SVA were highly correlated with ground truth values, with Pearson correlation coefficient (*R*) $>0.9$.

**Table 1:** Performance evaluation of the spinal radiographic parameters of the ensemble model.

Radiographic parameters	Ground truth	Parameter error^†^		Correlation analysis		Wilcoxon signed-rank test
	Mean (SD)	Mean (SD)	Median (IQR)	R	*p* value	*p* value
PI	51.6° (13.1°)	3.8° (5.7°)	1.9° (3.3°)	0.854	< 0.001*	0.587
SS	35.9° (12.1°)	3.5° (4.8°)	1.9° (3.0°)	0.875		0.578
PT	15.6° (11.0°)	1.1° (1.7°)	0.6° (0.9°)	0.983		0.430
LL	47.0° (17.4°)	5.1° (6.3°)	3.0° (4.7°)	0.885		0.145
SVA (mm)	25.2 (47.3)	1.9 (2.4)	1.0 (1.9)	0.998		0.402
CL	9.4° (17.3°)	6.6° (6.0°)	5.3° (6.4°)	0.864		0.636
T1S	24.4° (11.3°)	5.3° (5.1°)	3.8° (4.7°)	0.787		0.002*
cSVA (mm)	20.2 (13.4)	1.1 (1.4)	0.7 (1.0)	0.991		0.769
GTK	37.7° (14.4°)	6.6° (6.2°)	4.9° (6.5°)	0.798		0.029*
PTK	13.7° (9.7°)	6.7° (6.2°)	4.8° (6.5°)	0.558		0.013*
MTK	26.7° (13.5°)	5.4° (5.0°)	4.2° (5.6°)	0.840		0.726
L4SL	31.3° (11.5°)	4.3° (5.3°)	2.3° (4.2°)	0.810		0.133
LPA	8.3° (8.3°)	1.0° (1.7°)	0.5° (0.8°)	0.971		0.370
SSA	124.4° (14.8°)	3.4° (4.7°)	1.9° (3.0°)	0.919		0.480
GT	17.2° (15.0°)	1.2° (2.0°)	0.6° (1.0°)	0.988		0.636
TPA	13.1° (12.0°)	1.0° (1.6°)	0.5° (0.8°)	0.987		0.814
Barrey index	0.4 (3.1)	0.3 (1.5)	0.03 (0.1)	0.893		0.489
OD-HA	− 0.4° (3.9°)	0.1° (0.2°)	0.1° (0.1°)	0.999		0.167

*R* Pearson correlation coefficient; *SD* standard deviation; *IQR* interquartile range, *PI* pelvic incidence; *SS* sacral slope; *PT* pelvic tilt; *LL* lumbar lordosis; *SVA* sagittal vertical axis; *CL* cervical lordosis; *T1S* T1 slope; *cSVA* cervical sagittal vertical axis; *GTK* global thoracic kyphosis; *PTK* proximal thoracic kyphosis; *MTK* main thoracic kyphosis; *L4SL* L4-S1 lordosis; *LPA* lumbar pelvic angle; *SSA* spino-sacral angle; *GT* global tilt; *TPA* T1 pelvic angle; *OD-HA* odontoid hip axis.

*p value < 0.05.

^†^Absolute difference between the prediction and the ground truth.

Model performance varied for regional spinal parameters in different anatomic areas. For cervical parameters, the mean errors ranged from $1.1 \mathrm{mm}$ (cSVA) to $6.6^\circ$ (CL), and the median errors ranged from $0.7 \mathrm{mm}$ (cSVA) to $5.3^\circ$ (CL). For thoracic parameters, the errors were generally larger, with mean errors ranging from $5.4^\circ$ (MTK) to $6.7^\circ$ (PTK) and median errors ranging from $4.2^\circ$ (MTK) to $4.9^\circ$ (GTK). For lumbosacral parameters, the mean errors were $4.3^\circ$ (L4SL) and $1.0^\circ$ (LPA), and the median errors were $2.3^\circ$ (L4SL) and $0.5^\circ$ (LPA). Among the regional spinal parameters, significant differences were observed between predictions and the ground truth values for T1S, GTK, and PTK, with *p* < 0.05 in Wilcoxon signed-rank tests. The predicted cSVA and LPA had the closest correlations with ground truth values, with $R > 0.9$.

The proposed deep learning model performed well in predicting global spinopelvic parameters, with the mean errors ranging from $0.3$ (Barrey index) to $3.4^\circ$ (SSA), and the median errors ranged from $0.03$ (Barrey index) to $1.9^\circ$ (SSA). No statistical differences were present between the predictions and ground truth values, with all $p >0.05$ in Wilcoxon signed-rank tests. All the global spinopelvic parameters were strongly correlated with ground truth values, with all $R > 0.9$ except for the Barrey index ($R = 0.893$).

### Level of agreement between doctors and the deep learning model

**Figure 7:**
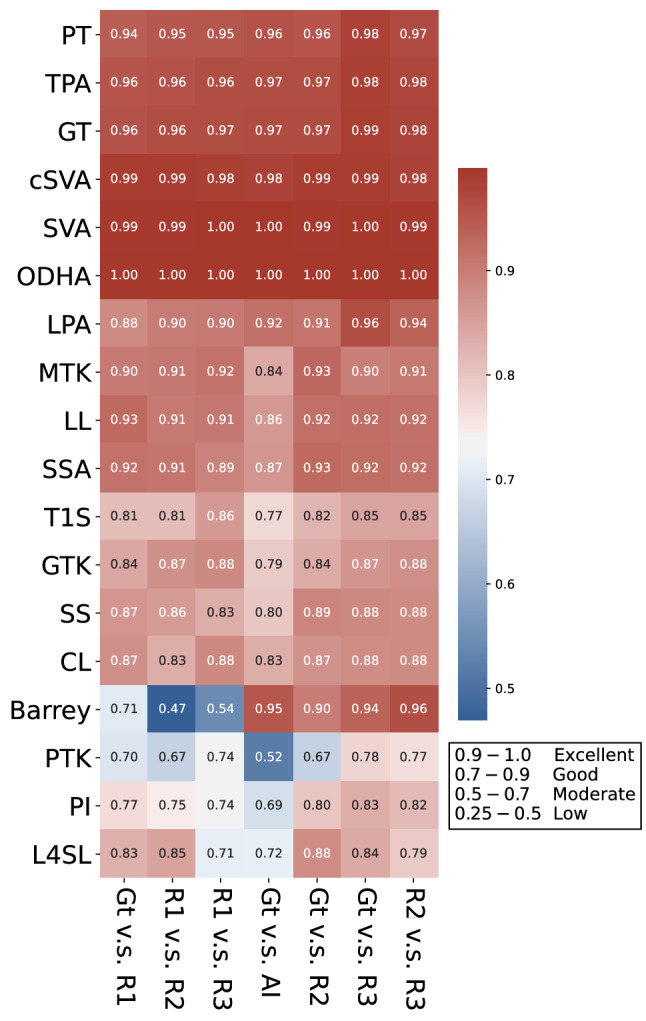
Heatmap of intraclass correlation coefficient (ICC) between human observers and the deep learning model. The interobserver reliability of three human observers (R1: junior resident; R2: spine fellow; R3: senior surgeon), the deep learning model (AI), and the ground truth values (Gt) was compared using the intraclass correlation coefficient (ICC). The ICC heatmap presented a data matrix, where colouring offers an overview of the numeric ICC differences for each radiographic parameter. Hierarchical cluster analysis was used to build a hierarchy of the ICC heatmap clusters. The deep learning model (AI) was capable of matching the reliability of human observers in 15/18 of the parameters.

Interobserver reliability comparisons between three human observers (a junior resident, spine fellow, and senior surgeon), the deep learning model, and the ground truth values were conducted using the intraclass correlation coefficient (ICC) for $90$ images within the dataset for interobserver reliability analysis, as described in “Datasets” of the Methods section. The ICC heatmap (Fig. 7) presented a data matrix, where colouring provided an overview of the numeric differences of the ICC for each radiographic parameter. Colour intensity represented the magnitude of ICC values, with a deeper red colour indicating a higher ICC and a deeper blue colour indicating a lower ICC. Reliability was categorised into four grades according to the ICC magnitude: excellent ($0.9-1.0$), high ($0.7-0.9$), moderate ($0.5-0.7$), and low ($0.25-0.5$). Hierarchical cluster analysis was used for comparing the overall performance of the deep learning model and the human observers. The sequences of the horizontal (interobserver comparisons) and vertical (radiographic parameters) axes were redistributed according to the trend of ICC magnitudes, such that higher interobserver reliability scores clustered towards the upper right corner and lower interobserver reliability scores clustered towards the lower left corner.

As can be observed in Fig. 7, the deep learning model achieved excellent reliability for the parameters of PT, TPA, GT, cSVA, SVA, and OD-HA compared with the reliability of the three human observers. For the parameters of LPA, MTK, LL, SSA, T1S, GTK, SS, and CL, the deep learning model and human observers achieved high reliability. Nonetheless, interobserver reliability was more divergent for the Barrey index, PTK, PI, and L4SL parameters; the colours of heatmap indicated variance. Compared with ground truth values, the deep learning model outperformed the human observers in the Barrey index but underperformed them in PTK, PI, and L4SL.

In brief, the deep learning model was capable of matching the reliability of human observers for 15 out of the 18 parameters.

## Discussion

Adult spinal deformity (ASD) is a debilitating condition present in 32%–68% of people older than 65 years^29,30^. The aetiologies involve a spectrum of diseases including de novo scoliosis, progressive adolescent idiopathic scoliosis, degenerative hyperkyphosis, and iatrogenic flat back deformity^31^. Radiographic assessment of the whole spine, including both hip joints, is recommended for the evaluation of sagittal balance in adult spinal deformity. Numerous studies have reported correlations of fundamental spinopelvic parameters with health-related quality of life metrics and the prognosis of ASD corrective surgeries^2,31–35^. Regional spinal parameters also play a prominent role in disease classification and preoperative planning^18,22,36,37^. Global spinopelvic parameters^23–27^, which facilitate the evaluation of the spinal curvature across more than two regions, enable overall assessment of sagittal balance without the influence of postural changes, body size differences, or regional compensating mechanisms for ASDs such as cervical hyperlordosis, thoracic hypokyphosis, and pelvic retroversion. In the clinical setting, manual measurement of all these parameters is time consuming and is sometimes influenced by interobserver variability^38–42^. In this study, we proposed a deep learning model demonstrating performance similar to those of human observers for 15 out of the 18 spinal sagittal radiographic parameters in different diseased spinal conditions.

Machine learning and deep learning have been widely applied for the automatic landmark localisation of spinal structures. With the aid of machine learning, the mean localisation errors of the vertebral body and intervertebral disc landmark identification on MRI have improved from 6.2 to 2.6 mm^43,44^. Deep learning has enabled further improvement of mean localisation errors for intervertebral discs to below 2 mm^45,46^. However, for pathologic spines or in the presence of metallic implants, mean localisation errors range between 6 and 8.5 mm when machine learning is applied^47–49^. Even with the assistance of deep learning, progress is limited, and mean localisation errors have marginally improved to between 6.9 and 9 mm in CT datasets of various spinal pathologies^50^. Our two-stage deep learning model was able to automatically localise 45 anatomic landmarks (24 vertebral centres and 21 specific landmarks) in a complex test dataset containing 400 randomly selected images of various spinal pathologies and different types of metallic implants. Model performance was the highest in the cervical area, with all median localisation errors lower than 3 mm. The occlusion of shoulder girdle structures may be the explanation for the wider distribution of localisation errors (Fig. 6) near C–T junctions. Landmarks in the thoracic area are difficult to predict, especially for patients with scoliosis in the midthoracic region. Overlapping of the vertebrae in severe scoliotic curves hinders clear recognition of each vertebral centre, and prediction under this condition typically requires repeat evaluations in both directions (cranial–caudal and caudal–cranial), even for experienced surgeons and radiologists. Unlike the thoracic region, the landmarks in lumbosacral area are not occluded by adjacent anatomic structures and can typically be clearly identified. In this study, the median localisation errors of the lumbosacral vertebral landmarks were all less than 3 mm. Although the median localisation errors were 3.39 mm and 2.75 mm, the recognition of bilateral femoral heads was worse than the recognition of the whole vertebral column. Partial occlusion by the contralateral femoral heads, poor contrast visualisation in the pelvic region, and the presence of metallic implants in the hip region may all affect the localisation of femoral head centres. Overall, our model performed better in the cervical and lumbosacral areas, but improvement is required for landmark identification in the thoracic and pelvic areas.

The two-stage deep learning model locates the 45 anatomic landmarks in a coarse-to-fine manner. Taking Fig. 3 as an example, the predicted heatmaps of landmarks in thoracic and thoracolumbar areas were less-localised and scattered along the spinal column between the anterior and posterior borders of the vertebrae in the first stage. These illegible landmarks then became more localised in the second stage of the model. On the other hand, the predicted heatmaps of landmarks in cervical and lumbosacral areas were extremely localised in the first stage already. Correct recognition of illegible landmarks is a challenge for the deep learning model under the following situations. First, landmarks with a higher degree of occlusion, e.g. thoracolumbar landmarks which were partially occluded by the rib cage, implants, or bone cement; Second, vertebral landmarks with high indistinguishability, e.g. T7 could not be determined easily because its structure and background were extremely similar to its adjacent levels. The presence of long tails of boxenplots in Fig. 6 was mainly caused by incorrect recognition of vertebral levels in these situations.

Several studies have also applied deep learning methods for automatic radiographic parameter prediction in individuals with spinal disorders. For Cobb angle measurement of adolescent idiopathic scoliosis, Wang et al*.*^7^ achieved circular mean absolute errors (CMAEs) of 7.81° and 6.26° in anteroposterior (AP) and lateral views, respectively, by using multi-view extrapolation net (MVE-Net). Wu et al*.*^9^ further improved CMAEs to 4.04°and 4.07° in AP and lateral views by using multi-view correlation network (MVC-Net), respectively. In addition, Galbusera et al*.*^8^ collected 493 biplanar EOS plain radiographs for model development and applied 78 convolutional neural networks to automatically recognise 78 landmarks. Radiographic parameters could be generated accordingly, including T4–T12 kyphosis, L1–L5 lordosis, Cobb angle of scoliosis, PI, PT, and SS, with the standard errors of the estimated parameters ranging from 2.7° (for PT) to 11.5° (for L1–L5 lordosis). Recently, Korez et al*.*^5^ developed a two-stage model for fully automatic measurement of sagittal spinopelvic parameters. In their model, RetinaNet was used for recognising specific areas such as C7, S1, and both femoral heads in the first stage. In the second stage, U-net was used for anatomic landmark detection, such as those in the centres and anterior and posterior corners within previously identified areas. With a small training dataset of 145 sagittal radiographs, they were able to achieve mean absolute errors ranging from $1.2^\circ \pm 1.2^\circ$(spinal tilt) to $5.5^\circ \pm 4.2^\circ$ (PI) for spinopelvic parameters, with significant correlations between manual measurements and their deep learning model. Nonetheless, the study included only images with degenerative pathologies with or without short segment fusions. The researchers also excluded images not automatically recognised in the first stage from the statistical analysis. Furthermore, the test datasets of the previous studies were relatively small, with fewer than 100 X-ray images. In our study, after generating the locations of 45 anatomic landmarks, 18 radiographic parameters were automatically calculated, most of which (15/18) had model performance comparable to those of human observers; the exceptions were PTK, PI, and L4SL (Fig. 7). The mean absolute errors ranged from $0.1^\circ \pm 0.2^\circ$(OD-HA) to $6.7^\circ \pm 6.2^\circ$ (PTK), and the median absolute errors ranged from 0.03 ± 0.1 (Barrey index) to $5.3^\circ \pm 6.4^\circ$ (CL; Table 1). Our deep learning approach achieved good performances in PT, cSVA, SVA, T1S, and SS, which can be determined using two anatomic landmarks and one reference line (either horizontal or vertical), as shown in Supplementary Fig. S1. The deep learning model also achieved good performances in radiographic parameters across a broad region (e.g. TPA, GT, OD-HA, SSA, and Barrey index) and across the mid-range region (≥ 5 levels; e.g. LPA, MTK, LL, GTK, CL). These parameters were determined by an angle between two crossed lines made of long-distant landmarks, and a small perturbation on these landmark coordinates would not increase the parameter error much. However, relatively low performances were observed in three parameters: PTK, PI, and L4SL. The possible explanations are as follows. The anatomic landmarks used for the measurement of PTK are across five levels in the upper thoracic area but usually blocked by the shoulder girdle (clavicle, scapula, humerus) and ribs. PI is a pelvic morphology parameter that requires four anatomic landmarks in the pelvis, including femoral heads, the landmarks with the most significant localization errors. Lastly, L4SL is a parameter whose measurement requires four anatomic landmarks across a relatively short distance, only two levels in the lumbosacral area.

The prevalence of adult spinal deformity has increased as the world gradually stepped into an ageing society^31^. Since whole-spine plain radiographs are the standard first-line examination, fast and accurate interpretation of the radiographic parameters through the deep learning approach can readily be applied in clinical practice. It is an arduous task to derive those spinopelvic parameters for a doctor in busy clinical settings. The deep learning model can reduce repetitive work and potentially improve clinical efficiency by reducing the time required to generate 18 radiographic parameters to nearly 1 second while maintaining a generally acceptable 5° error in most parameters. More importantly, this method can be used retrospectively on multi-institutional dataset to achieve an understanding of the distribution of spinal parameters at a populational level.

The main limitations of this study were the lack of external validation dataset. The current dataset contained images from only one medical centre. In addition, we excluded images with anatomic variance with anomalous vertebral numbering (more or fewer than 25) from the dataset. Therefore, our current deep learning model may not produce appropriate predictions for individuals with conditions that lead to anomalous vertebral numbering, such as lumbosacral transitional vertebrae or other congenital vertebral anomalies. To address this issue, it may be necessary to design an algorithm that determines the number and identities of vertebrae before localising landmarks. It is noteworthy that even for experienced surgeons, significant variability exists when measuring radiographic parameters in patients with anomalous vertebral numbering^51^. Two other aspects of our proposed model which still have room for improvement include the occurrence of incorrect level recognition and its relatively poor hip centre recognition. Improvement in these aspects shall be possible with a larger training dataset.

We demonstrated that with the aid of the proposed deep learning model, the accuracy of the automatic landmark localiser was within acceptable ranges for whole-spine lateral plain radiographs in a large test dataset consisting of images of various spinal pathologies. The model was capable of matching the reliability of human observers for 15 of the 18 parameters, and could potentially be applied in institutional practice to aid in clinical workflow and reduce research workload.

## Methods

This study was approved by the Institutional Review Board of Chang Gung Memorial Hospital, Taiwan (IRB No. 202000623B0) and carried out in accordance with the pertinent guidelines and regulations. The informed consent was obtained from all participants or their legal guardians prior to each clinical visit in the study.

### Datasets

A total of 2900 consecutive whole-spine lateral images collected in our hospital from January 2018 to April 2020 were reviewed and deidentified before data analyses. A senior radiologist screened the entire image dataset and excluded (1) 174 images with inadequate length that did not include either C2 dens or both femoral heads; (2) 294 images with anatomic variance in which the vertebral column contained fewer than or more than 25 vertebrae; and (3) 222 images with poor contrast, preventing identification of pelvic anatomic structures. After exclusion, a total of 2210 images were included and annotated in this study.

We further split 2210 images into three categories, namely scoliosis (1041 images), kyphosis (466 images), and implant (703 images), according to the disease aetiologies or the presence of one or more implants. The annotated 2210 images were then used to form the following datasets:

1. Dataset of children (120 images): we did not evaluate the model performance using images of children because the sacral ossification centre fused gradually from teenage to young adulthood. In this study, a senior spinal surgeon screened the annotated 2210 images and identified 120 images of them as totally unfused sacrum (aged less than 12 years old). This dataset of children was not used during inference but was included during training to enrich the diversity of training samples.
2. Test dataset (400 images): 2090 images were left after excluding 120 images of children. To evaluate the model performance, we randomly selected 400 images from 2090 images as the test dataset.
3. Dataset for interobserver reliability analysis (90 images): this dataset was obtained by selecting 30 images randomly from every image category (scoliosis, kyphosis and implant) of the test dataset. We used this dataset to analyse the agreement between three doctors and our deep-learning model.
4. Dataset for cross-validation (1690 images): after excluding the dataset of children and the test dataset, the rest images were for the procedure of fivefold cross-validation (1690 images). We used fivefold cross-validation for model training and selection.

### Labelling and classification of the datasets

We used a custom-written MATLAB GUI program for annotation of the whole-spine lateral images in Digital Imaging and Communications in Medicine (DICOM) format. The dataset of 2210 images underwent a three-stage peer-reviewed annotation process. The first-stage annotation was conducted by an annotation team comprising three junior orthopaedic residents and one senior orthopaedic resident. The annotated coordinates of all 45 anatomic landmarks were recorded accordingly. In the second stage, a spine fellow reviewed the first-stage annotations and classified the images into the following categories according to the disease aetiologies or the presence of metallic implants: scoliosis, kyphosis, and implant. Modifications were made by the spine fellow if a first-stage annotation was deemed incorrect. In the third stage, the data were reviewed and amended again by a senior spine surgeon. The finalised version of annotations was set as ground truth values and exported after integration by an engineer.

### Annotated landmarks and derived radiographic parameters

The 45 annotated landmarks were as follows: the dens centre of C2; anteroinferior and posteroinferior points of C2; anteroinferior, posterosuperior, and posteroinferior points of C7; anterosuperior and posterosuperior points of T1; the four corners of T5; anteroinferior and posteroinferior points of T12; anterosuperior and posterosuperior points of L1; anterosuperior and posterosuperior points of L4; anterior and posterior edges of the sacral endplate; both femoral head centres; and the vertebral centres from C2 to L5, as shown in Fig. 1. Within all the 45 landmarks, 21 specific landmarks and five vertebral centres were required to derive 18 spinopelvic parameters in this study. The other 19 vertebral centres were added to visualise the overall spinal alignment and could be used to analyse the curvature of the vertebral column in the future.

### Learning objective of the network

Given a 2D radiograph, the two-stage network aims to output 45 landmark coordinates: $\{({x}_{1}{y}_{1}), ({x}_{2}, {y}_{2}),.., ({x}_{45}, {y}_{45})\}$. For this regression task, we define the loss function of a single radiograph as follows:

1 $$L=-\frac{1}{45}{\sum }_{s=1}^{2}{\sum }_{k=1}^{45}[Wing\left({\widehat{x}}_{k}^{\left(s\right)}-{x}_{k}\right)+Wing\left({\widehat{y}}_{k}^{\left(s\right)}-{y}_{k}\right)]$$

where ${x}_{k}$ and ${y}_{k}$ are the $x$ and $y$ coordinates of the $k$th ground truth landmark; ${\widehat{x}}_{k}^{\left(s\right)}$ and ${\widehat{y}}_{k}^{\left(s\right)}$ are the $x$ and $y$ coordinates of the $k$th predicted landmark in the $s$th model stage; and $Wing( . )$ denotes the Wing loss function^52^:

2 $$Wing\left( x \right) = \left\{ {\begin{array}{*{20}l} {w{\text{ln}}\left( {1 + \frac{{\left| x \right|}}{\varepsilon }} \right)} \hfill & if\, |x| \,<\,w , \hfill \\ {\left| x \right| - w + w\ln \left( {1 + \frac{w}{\varepsilon }} \right)} \hfill & {otherwise} \hfill \\ \end{array} } \right.$$

where $w$ confines the range of non-linearity into $(-w, w)$ and $\epsilon$ limits the curvature of the non-linear region. The Wing loss function was initially adopted for facial landmark detection and was demonstrated to perform better than the commonly used Smooth L1 or L2 loss.

In addition, the two-stage network predicts the same regression target twice. We select the last-stage output as the final prediction because results of the last stage can generally be expected to be improved results of the former stage^53^.

### Gaussian or exponential heatmaps for heatmap regularisation

For each landmark $\left({x}_{k}, {y}_{k}\right)$, we predefine a distribution $P\left({x}_{k}, {y}_{k}\right)\in {\mathbb{R}}^{H\times W}$, which contains a small splotch centring at $\left({x}_{k}, {y}_{k}\right)$. Additional loss for heatmap regularisation (Jenson–Shannon entropy) is then considered to encourage that ${\widehat{P}}^{\left(k,s=2\right)}\sim {P}^{\left(k\right)}$. In our paper, we experiment with the following forms of $P\left({x}_{k}, {y}_{k}\right)$^6^:

3 $$\begin{aligned} G_{{ij}} \left( {x_{k} ,y_{k} ;\sigma } \right) & = C_{G} {\text{exp}}\left( { - \frac{{\left( {{\text{x}}_{{\text{i}}} - {\text{x}}_{k} } \right)^{2} + \left( {{\text{y}}_{{\text{j}}} - {\text{y}}_{k} } \right)^{2} }}{{2\sigma ^{2} }}} \right) \\ E_{{ij}} \left( {x_{k} ,y_{k} ;\sigma } \right) & = C_{E} exp\left( { - \frac{{{\text{log}}\left( 2 \right)\left( {\left| {x_{i} - x_{k} } \right| + \left| {y_{i} - y_{k} } \right|} \right)}}{{2\sigma }}} \right) \\ \end{aligned}$$

where $\sigma$ is a positive real number, ${G}_{ij}\left({x}_{k}, {y}_{k};\sigma \right)$ is a Gaussian, ${E}_{ij}\left({x}_{k}, {y}_{k};\sigma \right)$ is an exponential function, and ${C}_{G}$ and ${C}_{E}$ are the normalisation constants ensuring that ${\sum }_{ij}{ G}_{ij}\left({x}_{k}, {y}_{k}\right)=1$ and ${\sum }_{ij}{E}_{ij}\left({x}_{k}, {y}_{k}\right)=1$. These two functions are designed such that they reach the same half maxima at ${(x}_{k}\pm \sigma \sqrt{2\mathrm{log}2}, {y}_{k})$ and ${(x}_{k}, {y}_{k}\pm \sigma \sqrt{2\mathrm{log}2})$.

In the above setting, $\sigma$ is a hyperparameter of the model which controls the width of both Gaussian and exponential functions. In general, $\sigma$ has to be small enough so that the model can be encouraged to produce localised heatmaps in the 2^nd^ stage. However, it is inappropriate to have a too small value of $\sigma$. For example, in an extreme case (e.g. $\sigma <1)$, $P\left({x}_{k}, {y}_{k}\right)$ is extremely localised (almost only one pixel is non-zero), which means we unnecessarily encourage the network to be extremely certain about its estimation and the learning could become a challenge for the network.

### Extraction of landmark coordinates from heatmaps

The coordinate of the $k$th landmark in the $s$th model stage, $({\widehat{x}}_{k}^{\left(s\right)}, {\widehat{y}}_{k}^{\left(s\right)})$, is predicted using the differentiable spatial to numerical transform (DSNT) layer^12^ as follows:

4 $$\begin{aligned} \hat{x}_{k}^{{\left( s \right)}} & = \sum _{{i = 1}}^{H} \sum _{{j = 1}}^{W} {\text{X}}_{{{\text{ij}}}} \,Softmax\left( {\hat{P}_{{{\text{ij}}}}^{{\left( {k,{\text{s}}} \right)}} } \right) \\ \hat{y}_{k}^{{\left( s \right)}} & = \sum _{i}^{H} \sum _{j}^{W} Y_{{ij}\,} Softmax\left( {\hat{P}_{{ij}}^{{\left( {k,s} \right)}} } \right) \\ \end{aligned}$$

where $H$ and $W$ are the height and width of the input; ${X}_{ij}=\frac{2j-W-1}{W}\in \left[-1+\frac{1}{W}, 1-\frac{1}{W}\right]$ and ${Y}_{ij}=\frac{2i-H-1}{H}\in \left[-1+\frac{1}{H}, 1-\frac{1}{H}\right]$ are the predefined 2D matrices representing $x$ and $y$ coordinates; ${\widehat{P}}^{\left(\mathrm{k},\mathrm{s}\right)}\in {\mathbb{R}}^{H\times W}$ is the predicted 2D heatmap for landmark $\left({x}_{k}, {y}_{k}\right)$ in the $s$ ^th^ model stage; and $Softmax( . )$ denotes the operation of 2D $Softmax$, which was adopted to ensure the normalisation condition, ${\sum }_{ij}{\widehat{P}}_{ij}^{\left(p,s\right)}=1$.

DSNT can be interpreted as a layer which calculates the ‘expected values’ of landmark coordinates. To predict the landmark coordinates well, we expect each last-stage heatmap ${\widehat{P}}^{\left(\mathrm{k},\mathrm{s}=2\right)}$ to contain only one small splotch centring at $\left({x}_{k}, {y}_{k}\right)$.

### Weighted localisation error for model selection

To determine localisation quality, we can measure the Euclidean distance between the predicted and annotated landmark coordinates. The localisation error for landmark $i$ on image $j$ is defined as

5 $${\xi }_{i}^{\left(j\right)}= ||{\widehat{l}}_{i}^{\left(j\right)}-{l}_{i}^{\left(j\right)}|{|}_{2}$$

where ${\widehat{l}}_{i}^{\left(j\right)}\in {\mathbb{R}}^{2}$ (${l}_{i}^{\left(j\right)}\in {\mathbb{R}}^{2}$) is the predicted (annotated) landmark coordinates for landmark $i$ on image $j$.

We then consider the weight-averaged version of the localisation error for model selection. It is crucial to know that 24 of the 45 landmarks are annotated on vertebral centres, which have different extents of tolerable variability. For example, the size of C1 is much smaller than that of L5; hence, a 1-mm error for the L5 centre is less significant than a 1-mm error for the C1 centre. Considering the per-landmark error significance, we define the weighted localisation error for each landmark $i$ on image $j$ as follows:

6 $${\stackrel{\sim }{\xi }}_{i}^{\left(j\right)}=\frac{||{\widehat{l}}_{i}^{\left(j\right)}-{l}_{i}^{\left(j\right)}|{|}_{2}}{\mathrm{min}\left(\left\{ ||{l}_{i}^{\left(j\right)}-{l}_{k}^{\left(j\right)}|{|}_{2} | k=\mathrm{1,2},\dots ,i-1, i+1,\dots ,M\right\}\right)}$$

where $M$ is the number of landmarks to be localised. Based on the above explanation, an error of landmark $i$ on image $j$ is measured in the unit of the distance between the annotated landmark $i$ and its nearest annotated landmark neighbour. If ${\stackrel{\sim }{\xi }}_{i}^{\left(j\right)}=1$, we can conclude that the predicted location of landmark $i$ has the potential to reach to its nearest-neighbour landmark on image $j$. If ${\stackrel{\sim }{\xi }}_{i}^{\left(j\right)}\ll 1$, this indicates that landmark $i$ on image $j$ is well predicted and less likely to reach to the annotated locations of other landmarks. ${\stackrel{\sim }{\xi }}_{i}^{\left(j\right)}$ is a dimensionless quantity.

Due to the existence of highly occluded landmarks or ‘hard cases’, the error distributions are skewed in the experiments. Thus, instead of error mean, we compute the error median for each $j$th landmark as follows:

7 $${\stackrel{\sim }{\xi }}_{j}=Median\left\{{\stackrel{\sim }{\xi }}_{j}^{\left(1\right)}, {\stackrel{\sim }{\xi }}_{j}^{\left(2\right)},\dots ,{\stackrel{\sim }{\xi }}_{j}^{\left(N\right)}\right\}$$

where $N$ is the total number of images.

In this study, all landmark errors are expected to be maintained within a certain threshold, because many of them are directly related to one or more spinopelvic parameters. Thus, we use the maximum median error of landmarks for model selection, which is defined as:

8 $$\stackrel{\sim }{\xi }=Max\left\{{\stackrel{\sim }{\xi }}_{1},{\stackrel{\sim }{\xi }}_{2},\dots ,{\stackrel{\sim }{\xi }}_{M}\right\}$$

### Model selection

Two hyperparameters are fine-tuned to obtain the optimal model, namely the heatmap type for regularisation (Gaussian, exponential, or none) and the width of heatmap for regularisation ($\sigma \in [{d}_{\sigma }, {d}_{\sigma }/2, {d}_{\sigma }/4]$, if applicable). ${d}_{\sigma }$ is obtained using the following heuristics. We estimate the mean distance between consecutive vertebral centres, which is 41.2 (pixels). We then assume that a suitable Gaussian splotch should be confined approximately within this distance. Thus, we require ${6d}_{\sigma }=41.2$ (pixels) and obtain ${d}_{\sigma }=6.9$.

After fivefold cross-validation of the dataset (1690 radiographs), we observed that the model trained with heatmap regularisation (exponential function, $\sigma ={d}_{\sigma }/4$) and the model trained without heatmap regularisation have the highest performance. These two models achieved $\stackrel{\sim }{\xi }=0.48$ ($IQR=0.45$) and $\stackrel{\sim }{\xi }=0.48$ ($IQR=0.47$), respectively.

### Model ensemble

An ensemble of the two optimal models was created to boost predictive performance. The ensemble was obtained by averaging (unweighted) their predictions.

### Training details

All radiographs were downsized to $864\times 382$ px and then padded to $864\times 480$ px in the pre-processing pipeline. During training, several operations of data augmentation were applied to prevent the deep learning model from adapting to images of certain scales, orientations, and types of noise. The operations used were random scaling (scale ranging from $0.8$ to $1.0$), random rotation (angle ranging from $-{30}^{^\circ }$ to ${30}^{^\circ }$), and random Gaussian blur (strength ranging from $0$ to $0.1$). We used the Adam optimiser for loss minimisation during training. For all training experiments, we trained the model for 120 epochs. However, if the model’s performance did not improve within 20 epochs, we ended the training early. The learning rate of the optimiser was set to 0.005 at the beginning and was reduced to 0.0005 at epoch 100. The model was implemented and trained using TensorFlow 2.1.0. and Horovod 0.18.2. We used six NVIDIA Tesla V100 GPUs for training. The batch size was set to 18 (3 images per GPU). We used cross-GPU batch normalisation^54^ so that both the mean and variance were estimated using tensors scattered to all GPUs. Using TensorFlow, we turned on automatic mixed-precision training^55^ for faster training and reduced GPU memory use.

### Statistical analysis

Our deep learning approach was evaluated using the test dataset (400 images) for landmark localisation and parameter estimation. Due to the non-normal distribution of localisation errors, we used boxenplots to visualise error distributions. For parameter estimation, median parameter errors with IQR are presented in addition to mean parameter errors with SD. Pearson correlation coefficients were used to evaluate the correlations of all predicted radiographic parameters and the ground truth values for the corresponding parameters. Wilcoxon signed-rank tests were used to evaluate the numerical differences between the deep learning model and the ground truth values. A $p$ value $<0.05$ was considered significant.

The ICC was used to evaluate interobserver reliability between three human observers (a junior resident, spine fellow, and senior surgeon), the deep learning model, and the ground truth values using the dataset for interobserver reliability analysis (90 images). Reliability was classified into four grades according to the magnitude of the ICC: excellent ($0.9-1.0$), high ($0.7-0.9$), moderate ($0.5-0.7$), and low ($0.25-0.5$). The ICC data matrix was illustrated in the heatmap, where colouring provided an overview of the numeric ICC differences for each radiographic parameter. Hierarchical cluster analysis was used to build a hierarchy of the ICC heatmap clusters. As a result, the sequences of the horizontal (interobserver reliability) and vertical (radiographic parameters) axes were redistributed according to the trend of ICC magnitudes, such that higher ICC values cluster towards the upper-right corner and lower ICC values cluster towards the lower-left corner.

## Supplementary Information

Supplementary Information.

## Data Availability

The training and test datasets generated for this study contain protected patient information. Some data may be available from the corresponding author for research purposes upon reasonable request.

## References

[1] Dubousset J, Weinstein SL (1994). The Pediatric Spine: Principles and Practice.

[2] Le Huec JC, Thompson W, Mohsinaly Y, Barrey C, Faundez A (2019). Sagittal balance of the spine. Eur. Spine. J..

[3] Chwialkowski MP, Shile PE, Pfeifer D, Parkey RW, Peshock RM (1991). Automated localization and identification of lower spinal anatomy in magnetic resonance images. Comput. Biomed. Res..

[4] Peng Z, Zhong J, Wee W, Lee JH (2005). Automated vertebra detection and segmentation from the whole spine MR images. Conf. Proc. IEEE Eng. Med. Biol. Soc..

[5] Korez R, Putzier M, Vrtovec T (2020). A deep learning tool for fully automated measurements of sagittal spinopelvic balance from X-ray images: Performance evaluation. Eur. Spine J..

[6] Weng C-H (2019). Artificial intelligence for automatic measurement of sagittal vertical axis using ResUNet framework. J. Clin. Med..

[7] Wang L (2019). Accurate automated Cobb angles estimation using multi-view extrapolation net. Med. Image Anal..

[8] Galbusera F (2019). Fully automated radiological analysis of spinal disorders and deformities: A deep learning approach. Eur. Spine J..

[9] Wu H, Bailey C, Rasoulinejad P, Li S (2018). Automated comprehensive adolescent idiopathic scoliosis assessment using MVC-Net. Med. Image Anal..

[10] Al Arif S, Knapp K, Slabaugh G (2018). Fully automatic cervical vertebrae segmentation framework for X-ray images. Comput. Methods Programs Biomed..

[11] Chen, Y. *et al.* In *Proceedings of the IEEE conference on computer vision and pattern recognition* 7103–7112.

[12] Nibali, A., He, Z., Morgan, S. & Prendergast, L. Numerical coordinate regression with convolutional neural networks. *arXiv preprint *(2018).

[13] Stagnara P (1982). Reciprocal angulation of vertebral bodies in a sagittal plane: Approach to references for the evaluation of kyphosis and lordosis. Spine.

[14] Duval-Beaupère G, Schmidt C, Cosson P (1992). A Barycentremetric study of the sagittal shape of spine and pelvis: The conditions required for an economic standing position. Ann. Biomed. Eng..

[15] Jackson RP, McManus AC (1994). Radiographic analysis of sagittal plane alignment and balance in standing volunteers and patients with low back pain matched for age, sex, and size. A prospective controlled clinical study. Spine.

[16] Legaye J, Duval-Beaupère G, Hecquet J, Marty C (1998). Pelvic incidence: A fundamental pelvic parameter for three-dimensional regulation of spinal sagittal curves. Eur. Spine J..

[17] Hardacker JW, Shuford RF, Capicotto PN, Pryor PW (1997). Radiographic standing cervical segmental alignment in adult volunteers without neck symptoms. Spine.

[18] Le Huec JC, Demezon H, Aunoble S (2015). Sagittal parameters of global cervical balance using EOS imaging: Normative values from a prospective cohort of asymptomatic volunteers. Eur. Spine J..

[19] Bunnell WP (1988). The natural history of idiopathic scoliosis. Clin. Orthop. Relat. Res..

[20] Lenke LG (2005). Lenke classification system of adolescent idiopathic scoliosis: Treatment recommendations. Instr. Course Lect..

[21] Yilgor C (2017). Relative lumbar lordosis and lordosis distribution index: Individualized pelvic incidence-based proportional parameters that quantify lumbar lordosis more precisely than the concept of pelvic incidence minus lumbar lordosis. Neurosurg Focus.

[22] Protopsaltis TS (2018). The lumbar pelvic angle, the lumbar component of the T1 pelvic angle, correlates with HRQOL, PI-LL mismatch, and it predicts global alignment. Spine.

[23] Barrey C, Jund J, Noseda O, Roussouly P (2007). Sagittal balance of the pelvis-spine complex and lumbar degenerative diseases. A comparative study about 85 cases. Eur. Spine J..

[24] Boissière L (2017). Global tilt and lumbar lordosis index: Two parameters correlating with health-related quality of life scores-but how do they truly impact disability?. Spine J.

[25] Protopsaltis T (2014). TheT1 pelvic angle, a novel radiographic measure of global sagittal deformity, accounts for both spinal inclination and pelvic tilt and correlates with health-related quality of life. J. Bone Joint Surg. Am..

[26] Barrey C, Roussouly P, Le Huec JC, D'Acunzi G, Perrin G (2013). Compensatory mechanisms contributing to keep the sagittal balance of the spine. Eur Spine J.

[27] Amabile C (2016). A new quasi-invariant parameter characterizing the postural alignment of young asymptomatic adults. Eur. Spine J..

[28] Heike H, Wickham H, Kafadar K (2017). Letter-Value Plots: Boxplots for large data. J. Comput. Graph. Stat..

[29] Kebaish KM, Neubauer PR, Voros GD, Khoshnevisan MA, Skolasky RL (2011). Scoliosis in adults aged forty years and older: Prevalence and relationship to age, race, and gender. Spine.

[30] Schwab F (2005). Adult scoliosis: Prevalence, SF-36, and nutritional parameters in an elderly volunteer population. Spine.

[31] Diebo BG (2019). Adult spinal deformity. Lancet.

[32] Iyer S (2018). Sagittal spinal alignment in adult spinal deformity: An overview of current concepts and a critical analysis review. JBJS Reviews.

[33] Schwab FJ (2013). Radiographical spinopelvic parameters and disability in the setting of adult spinal deformity: A prospective multicenter analysis. Spine.

[34] Gussous Y, Theologis AA, Demb JB, Tangtiphaiboontana J, Berven S (2018). Correlation between lumbopelvic and sagittal parameters and health-related quality of life in adults with lumbosacral spondylolisthesis. Global Spine J..

[35] Glassman SD (2005). The impact of positive sagittal balance in adult spinal deformity. Spine.

[36] Ling FP (2018). Which parameters are relevant in sagittal balance analysis of the cervical spine? A literature review. Eur. Spine J..

[37] Lenke LG (2007). The Lenke classification system of operative adolescent idiopathic scoliosis. Neurosurg. Clin. N. Am..

[38] Marques C (2020). Accuracy and reliability of X-ray measurements in the cervical spine. Asian Spine J..

[39] Chung NS, Jeon CH, Lee HD, Won SH (2017). Measurement of spinopelvic parameters on standing lateral lumbar radiographs: Validity and reliability. Clin. Spine Surg..

[40] Kyrölä KK (2018). Intra- and interrater reliability of sagittal spinopelvic parameters on full-spine radiographs in adults with symptomatic spinal disorders. Neurospine.

[41] Lafage R (2015). Validation of a new computer-assisted tool to measure spino-pelvic parameters. Spine J.

[42] Gupta M (2016). Dedicated spine measurement software quantifies key Spino-Pelvic parameters more reliably than traditional picture archiving and communication systems tools. Spine.

[43] Schmidt S (2007). Spine detection and labeling using a parts-based graphical model. Inf. Process Med. Imaging.

[44] Oktay AB, Akgul YS (2011). Localization of the lumbar discs using machine learning and exact probabilistic inference. Med. Image Comput. Comput. Assist. Interv..

[45] Chen C (2015). Localization and segmentation of 3D Intervertebral discs in MR images by data driven estimation. IEEE Trans. Med. Imaging.

[46] Forsberg D, Sjöblom E, Sunshine JL (2017). Detection and labeling of vertebrae in MR images using deep learning with clinical annotations as training data. J. Digit. Imaging.

[47] Glocker B, Feulner J, Criminisi A, Haynor DR, Konukoglu E (2012). Automatic localization and identification of vertebrae in arbitrary field-of-view CT scans. Med. Image Comput. Comput. Assist Interv..

[48] Glocker B, Zikic D, Konukoglu E, Haynor DR, Criminisi A (2013). Vertebrae localization in pathological spine CT via dense classification from sparse annotations. Med. Image Comput. Comput. Assist. Interv..

[49] Lootus M, Kadir T, Zisserman A, Yao J, Klinder T, Li S (2015). Computational Methods and Clinical Applications for Spine Imaging.

[50] Yang, D. *et al.* In *Medical Image Computing and Computer Assisted Intervention − MICCAI 2017.* (eds Maxime Descoteaux *et al.*) 498–506 (Springer International Publishing).

[51] Khalsa AS (2018). Variability in assessing spinopelvic parameters with lumbosacral transitional vertebrae: Inter- and intraobserver reliability among spine surgeons. Spine.

[52] Feng, Z.-H., Kittler, J., Awais, M., Huber, P. & Wu, X.-J. In *Proceedings of the IEEE Conference on Computer Vision and Pattern Recognition* 2235–2245.

[53] Newell, A., Yang, K. & Deng, J. *European Conference on Computer Vision* 483–499 (Springer).

[54] Peng, C. *et al.* In *Proceedings of the IEEE Conference on Computer Vision and Pattern Recognition* 6181–6189.

[55] Micikevicius, P. *et al.* Mixed precision training. *arXiv preprint *(2017).

