# Supplementary material for: Deep learning approach for automatic landmark detection and alignment analysis in whole-spine lateral radiographs
Source: Sci Rep. 2021 Apr 7;11:7618. doi: 10.1038/s41598-021-87141-x (PMC8027006; doi:10.1038/s41598-021-87141-x)
Supplement: Supplementary file 1 — Supplementary Information. [file 41598_2021_87141_MOESM1_ESM.docx]

**Automatic landmark detection and alignment analysis in whole-spine lateral radiographs: a deep learning approach**

Yu-Cheng Yeh, MD^1,†^, Chi-Hung Weng, MSc^2,†^, Yu-Jui Huang, MD^1^,

Chen-Ju Fu, MD^3^, Tsung-Ting Tsai, MD, PhD^1,^*, Chao-Yuan Yeh, MD^2,^*

^1^ Department of Orthopaedic Surgery, Spine Division, Bone and Joint Research Center, Chang Gung Memorial Hospital and Chang Gung University College of Medicine, Taoyuan, Taiwan

^2^ aetherAI Co., Ltd., Taipei, Taiwan

^3^ Department of Medical Imaging and Intervention, Chang Gung Memorial Hospital and Chang Gung University College of Medicine, Taoyuan, Taiwan

^†^ YC Yeh and CH Weng contributed equally to this work.

* TT Tsai and CY Yeh contributed equally to this work.

There are no conflicts of interest to disclose.

In consideration of Springer Nature 's publication of this article, the authors hereby transfer, assign, and otherwise convey all copyright ownership worldwide to Springer Nature.

IRB No.: 202000623B0

*Corresponding author:

Chao-Yuan Yeh

aetherAI Co., Ltd., Taipei, Taiwan

9F., No.3-2, Yuanqu St., Nangang Dist., Taipei City 115, Taiwan

Tel: +886-2-27856892 #10

Email: joeyeh@aetherai.com

**Supplementary Figure Legends**

**
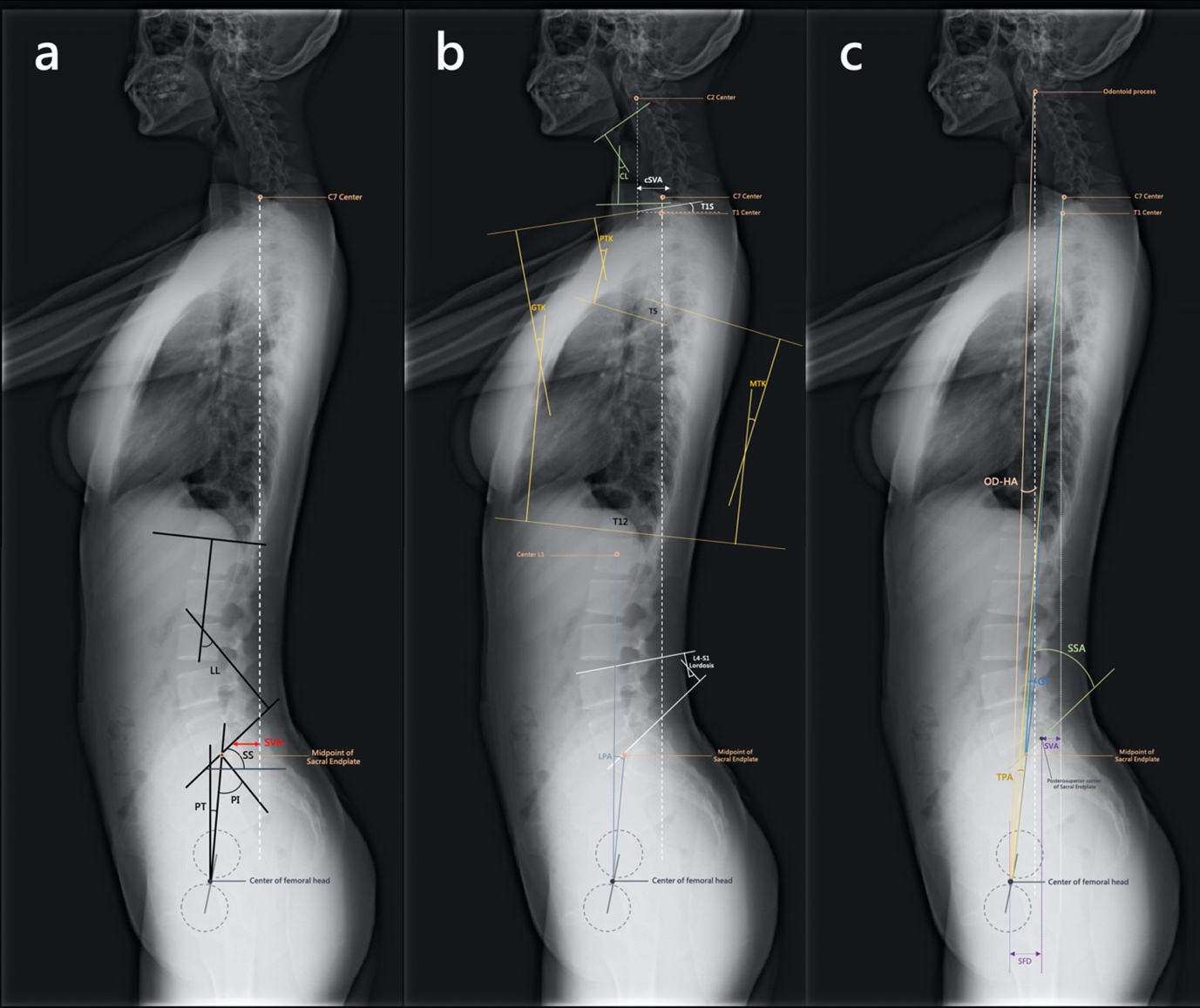
Supplementary Figure 1. Illustrations of 18 radiographic parameters**

a: Fundamental spinopelvic parameters: pelvic incidence (PI), sacral slope (SS), pelvic tilt (PT), lumbar lordosis (LL), and sagittal vertical axis (SVA).

b: Regional spinal parameters: cervical lordosis (CL), T1 slope (T1S), cervical SVA (cSVA), global thoracic kyphosis (GTK), T1–T5 proximal thoracic kyphosis (PTK), T5–T12 main thoracic kyphosis (MTK), L4-S1 lordosis (L4SL), and lumbar pelvic angle (LPA).

c: Global spinopelvic parameters: spino-sacral angle (SSA), global tilt (GT), T1-pelvic angle (TPA), C7 plumb line/sacrofemoral distance ratio (C7/SFD ratio or Barrey index), and odontoid hip axis (OD-HA).
